# Appendiceal low-grade pseudomyxoma peritonei recurrence with splenic invasion and parastomal hernia

**DOI:** 10.3389/fsurg.2024.1484812

**Published:** 2024-11-21

**Authors:** Qi Liu, Jie Jiao, Guanying Yu, Peiming Guo, Chengzhen Li

**Affiliations:** ^1^ School of Clinical Medicine, Shandong Second Medical University, Weifang, Shandong, China; ^2^ Department of Gastrointestinal Surgery, Central Hospital Affiliated to Shandong First Medical University, Jinan, China

**Keywords:** PMP, CRS, HIPEC, splenic invasion metastasis, parastomal hernia

## Abstract

Recurrence of low-grade appendiceal pseudomyxoma peritonei (PMP) with splenic invasion and parastomal hernia is exceptionally rare. We present a 47-year-old female with recurrent PMP, four years post-cytoreductive surgery (CRS) and hyperthermic intraperitoneal chemotherapy (HIPEC). She presented with abdominal distension, splenic invasion, and parastomal hernia. Imaging revealed extensive peritoneal and pelvic metastases, splenic lesions, and parastomal hernia. Intraoperative findings confirmed widespread pseudomyxoma, involving the spleen and diaphragm. She underwent CRS, splenectomy, tumor resection, adhesiolysis, partial colectomy, hernia repair, and diaphragmatic reconstruction, followed by intraoperative HIPEC. Despite postoperative complications, the patient recovered well with no recurrence over 20 months. This case underscores the challenges of managing recurrent PMP with splenic metastases and parastomal hernias, highlighting the importance of multidisciplinary collaboration and personalized therapeutic strategies.

## 1 Introduction

Pseudomyxoma peritonei (PMP) is a rare clinical syndrome characterized by the accumulation of abundant mucin within the abdominal cavity, involving the peritoneum, omentum, and pelvic organs (1). It typically originates from the rupture of mucinous tumors of the appendix. Due to its unique biological behavior, PMP primarily spreads intraperitoneally, with lymphatic and hematogenous metastases being relatively rare (2). Consequently, reports of PMP metastasizing to solid organs like the spleen are scarce, and the literature documenting such cases is very limited.

Cytoreductive surgery (CRS) combined with hyperthermic intraperitoneal chemotherapy (HIPEC) has become the standard treatment for PMP. The aim is to surgically remove the tumors deposits and administer intraperitoneal chemotherapy to minimize the number of residual tumor cells (3). Although CRS combined with HIPEC can effectively control the progression of PMP, its recurrence rate remains high. Recurrent PMP often manifests as the re-emergence of localized or systemic disease, potentially accompanied by symptoms such as abdominal pain, ascites, small bowel obstruction, malnutrition, intra-abdominal adhesions, and gastrointestinal bleeding (4, 5).

 Parastomal hernia is an uncommon complication of recurrent PMP, primarily driven by increased intra-abdominal pressure from high-volume mucinous tumor recurrence. This pressure exacerbates weakness at the stoma site, predisposing to herniation. Following CRS and HIPEC, the risk of abdominal wall hernias increases due to tissue trauma and compromised abdominal wall integrity.

Here, we report a case of recurrent PMP four years after undergoing CRS combined with HIPEC treatment. The patient developed rare splenic invasion and concurrent parastomal hernia formation, adding complexity to the subsequent surgical intervention. Through this case presentation, we aim to explore the biological characteristics, recurrence patterns, and implications for treatment strategies of PMP, providing valuable insights for clinical practice.

## 2 Case report

In July 2022, a 47-year-old female presented with a six-month history of abdominal distension. Four years prior, she had undergone CRS combined with open HIPEC for appendiceal PMP, which included total hysterectomy, bilateral salpingo-oophorectomy, and right hemicolectomy. Her preoperative PCI score was 30, and postoperative PCI was reduced to 6. Thermal ablation was applied to peritoneal tumors, with residual tumors located in the pelvic cavity and the diaphragm. Cytoreduction was incomplete, classified as CC2. Postoperatively, the patient developed an intraperitoneal infection leading to an anastomotic dehiscence of the ileocolic anastomosis, necessitating a double-barrel ileostomy and colostomy. Three months later, the colostomy was reversed, leaving the ileostomy in place. Systemic chemotherapy was not administered, as it was not indicated for her low-grade PMP.

**Figure 1:**
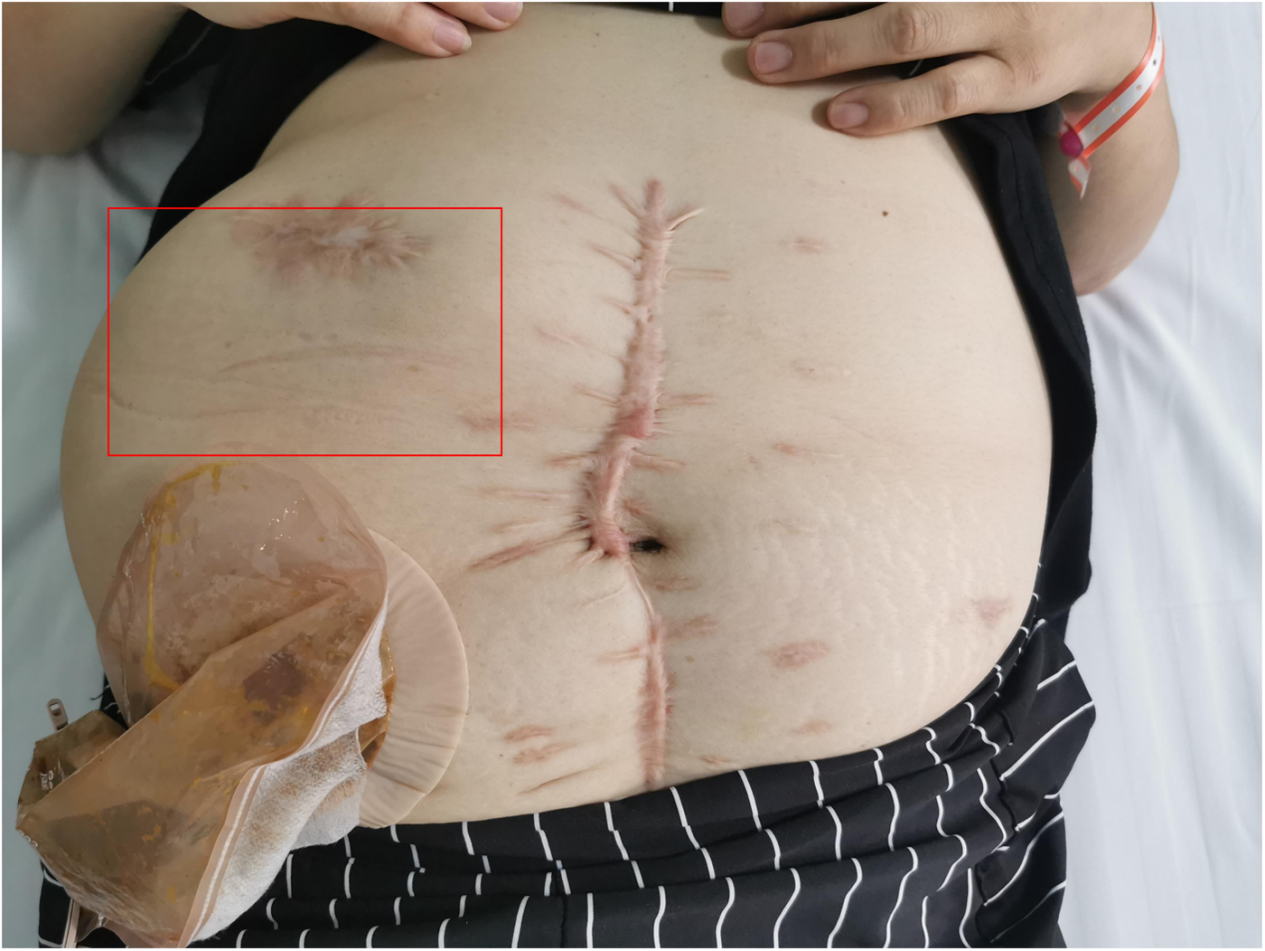
The right abdomen demonstrates a prominent protrusion (highlighted in red).

**Figure 2:**
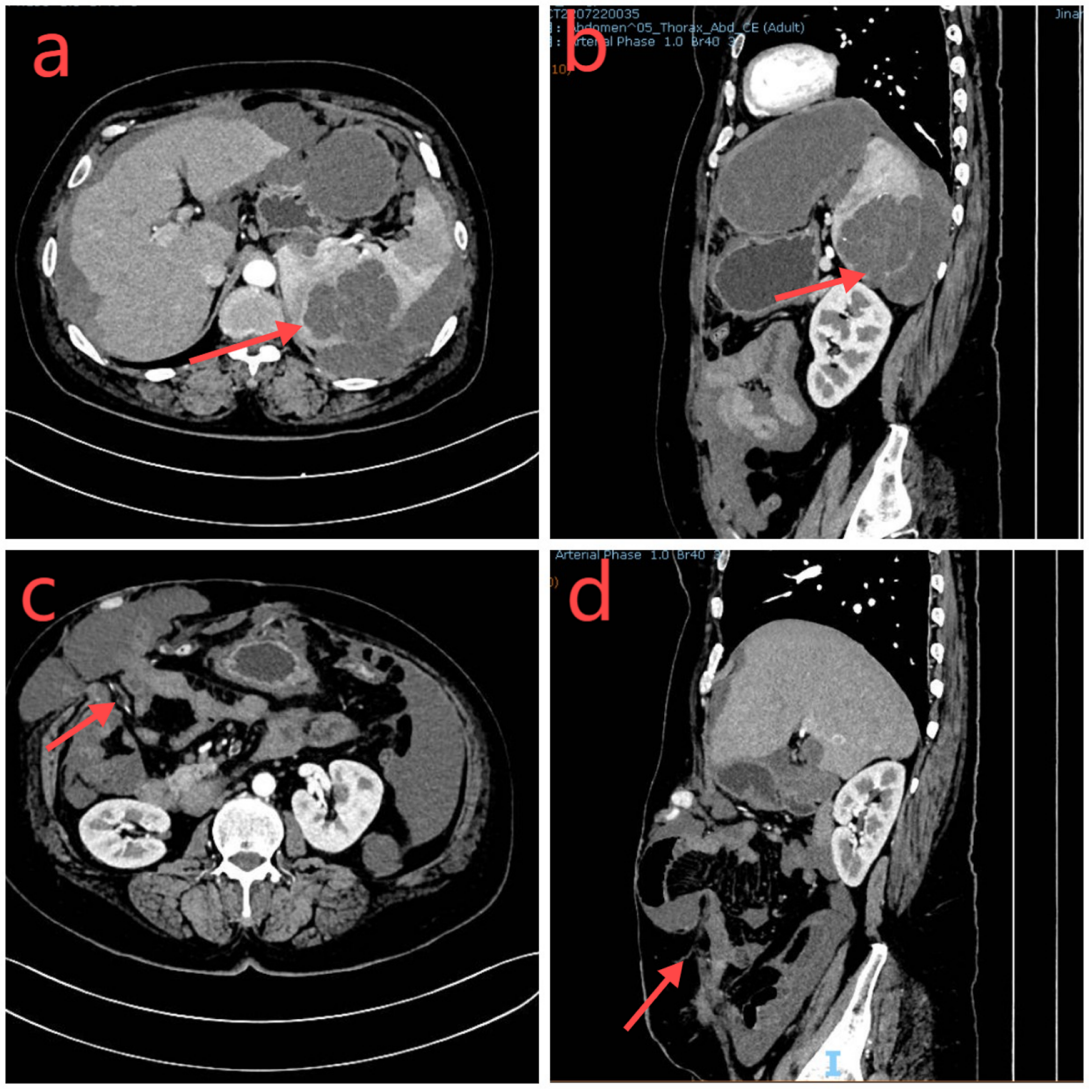
**(a,b)** The abdominal CT axial and sagittal sections demonstrate a low-density occupying lesion in the spleen (red arrow). **(c,d)**: Adjacent to the original colostomy site, there is a herniation of the small intestine and surrounding tissues (highlighted in red).

**Figure 3:**
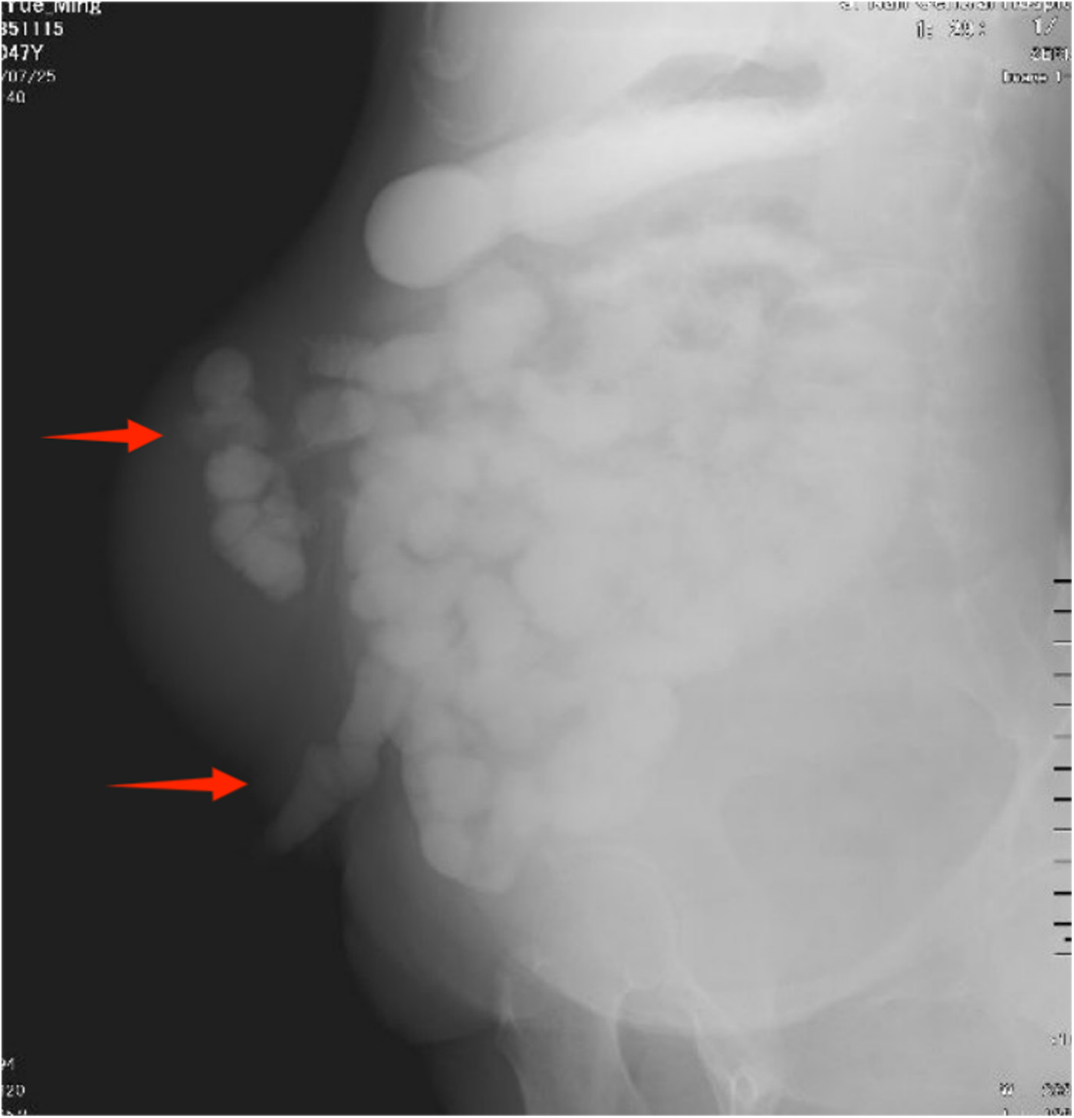
The gastrointestinal contrast study suggests hernia contents containing intestinal loops.

Physical examination revealed a stoma in the right lower abdomen, significant bulging on the right side of the abdomen (Figure 1), palpable hard masses, and shifting dullness. Serum carcinoembryonic antigen (CEA) levels elevated to 45.02 ng/ml, and CA19-9 levels markedly increased to 68.08 U/ml, Enhanced CT imaging revealed widespread low-density lesions in the bilateral subdiaphragmatic, hepatic, and pelvic regions, heterogeneous density lesions in the spleen, and diffuse thickening of the peritoneum in some areas. A mass was observed beside the original right upper abdominal colostomy, with protrusion of the small intestine into the subcutaneous layer of the abdominal wall. (Figure 2) Subsequent gastrointestinal contrast imaging confirmed these findings (Figure 3), consistent with the clinical features of progressive pseudomyxoma peritonei with extensive pelvic metastases, splenic invasion, and parastomal hernia.

**Figure 4:**
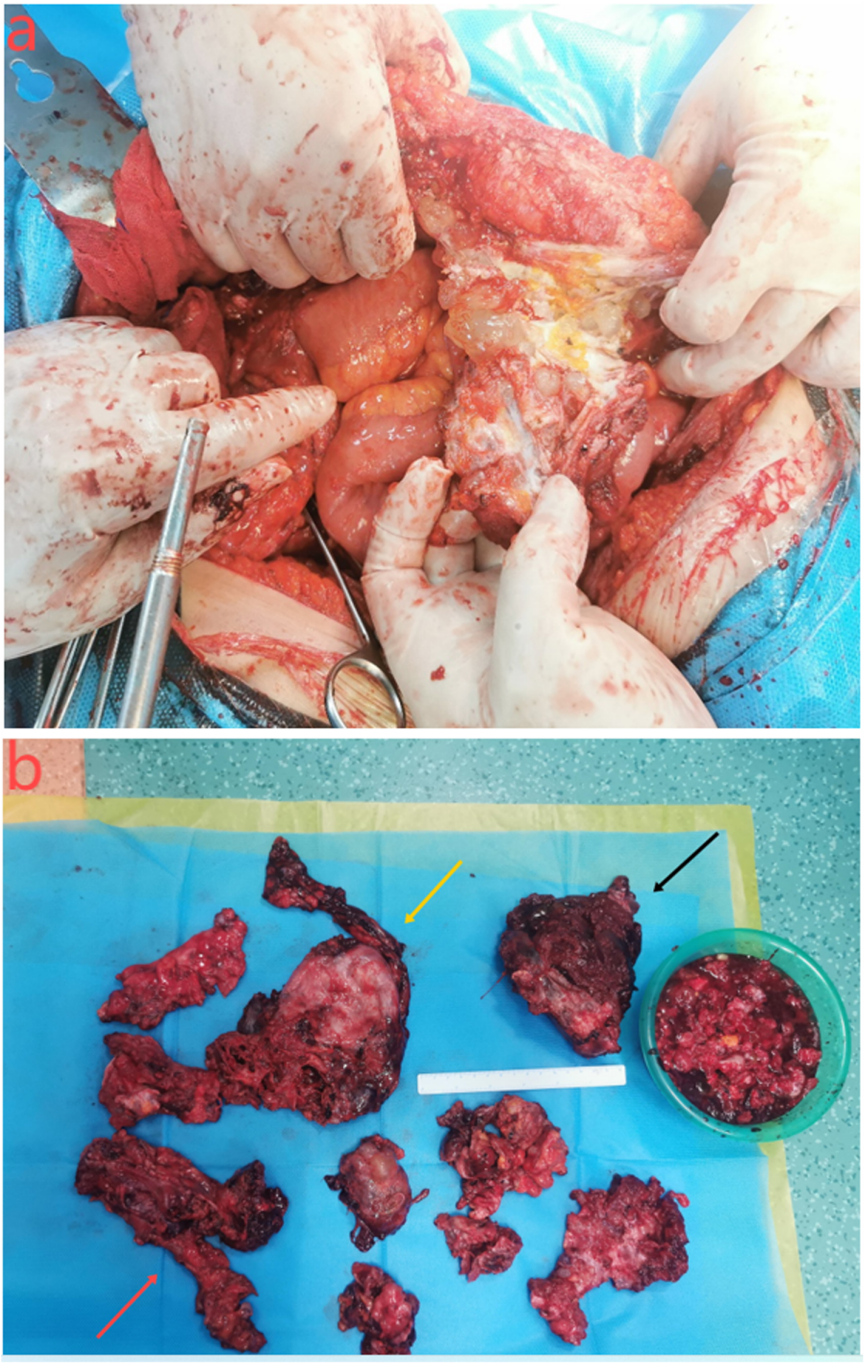
**(a)** the contents of the hernia are the small intestine and tumors. **(b)** Surgical resection specimen showing adhesion between the ileum and ascending colon (red arrow); large peritoneal tumor (yellow arrow); splenomegaly (black arrow).

During the surgery, extensive upper abdominal adhesions were identified, and an 8 cm hernia sac was observed adjacent to the original right ascending colon stoma (Figure 4a). Approximately 2,000 ml of mucinous, blood-tinged ascites was present. There was widespread deposition of mucinous tumor between the peritoneum, liver, and diaphragm, as well as within the hepatic round ligament. Portions of the peritoneum displayed thickened, “pancake-like” deposits, measuring up to 10 cm in diameter. Small, gravel-like mucinous deposits were also found on the greater omentum. Metastatic nodules were noted between the spleen and left upper abdominal wall, with significant infiltration into the diaphragmatic peritoneum. The PCI at laparotomy was calculated to be 24.

**Figure 5:**
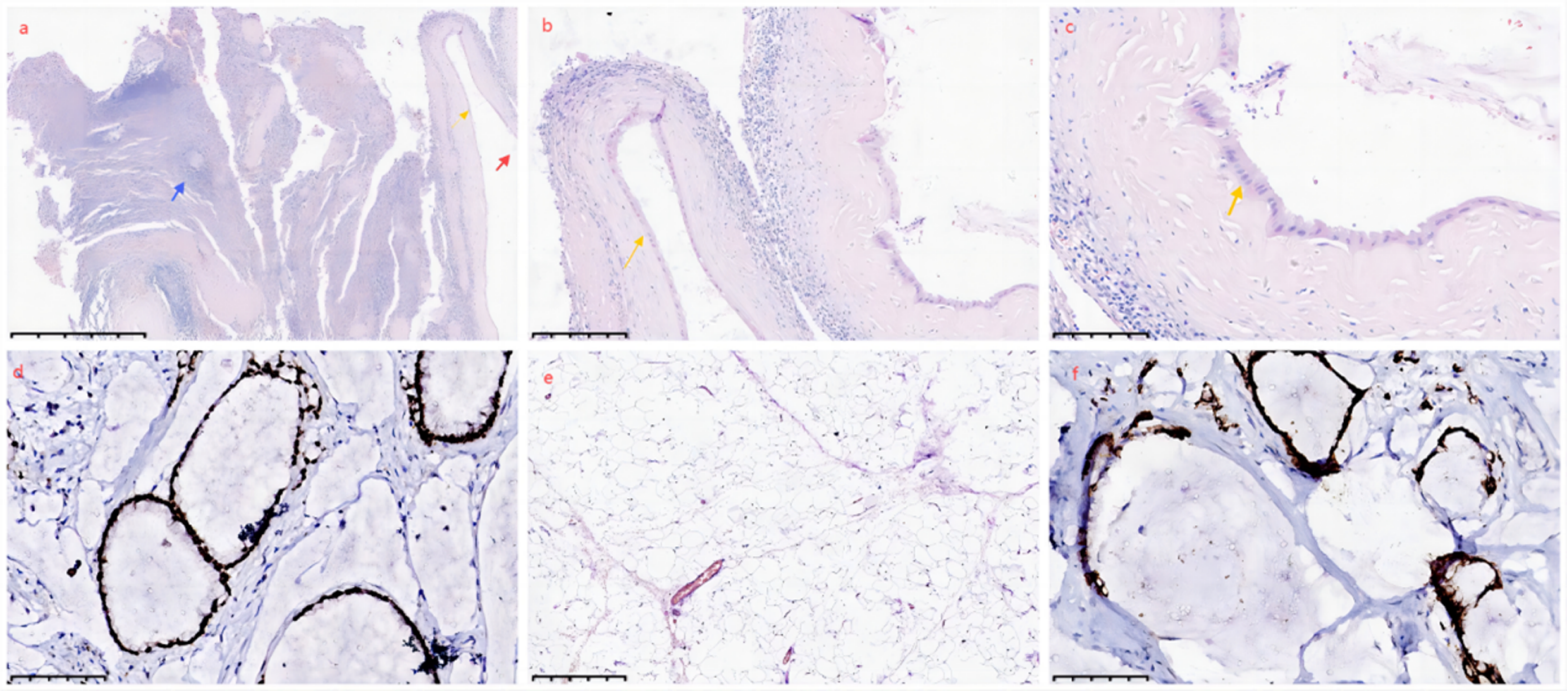
Spleen and immunohistochemistry: under the light microscope, cattered cystic lesions are observed in the splenic tissue, filled with mucoid material, surrounded by chronic inflammatory reaction. Splenic white pulp (blue arrow), single-layered tumor cells (yellow arrow), mucin (red arrow). **(a)** HE 30x, **(b)** HE 100x, **(c)** HE 200x, **(d)** Positive expression of MUC2. **(e)** Mucinous tissue. HE 100x. **(f)** Positive expression of CDX2.

Cytoreductive surgery was performed, including splenectomy, peritoneal tumor resection, adhesiolysis, and partial descending colon resection (Figure 4b), resulting in a postoperative peritoneal cancer index (PCI) score of 6 and a completeness of cytoreduction (CC) score of 1. Concurrently, parastomal hernia repair (using UPA31015 mesh, manufactured by Johnson & Johnson International c/o European Logistics Centre) composed of approximately equal amounts of absorbable monofilament polyglecaprone-25 fibers and non-absorbable polypropylene monofilament fibers. Left diaphragmatic repair were conducted, with closed thoracic drainage, followed by open HIPEC treatment. Pathological confirmation revealed a low-grade mucinous tumor of the appendix, with immunohistochemistry showing (spleen) CDX2+, Muc2+, Muc5+ (Figure 5).

**Figure 6:**
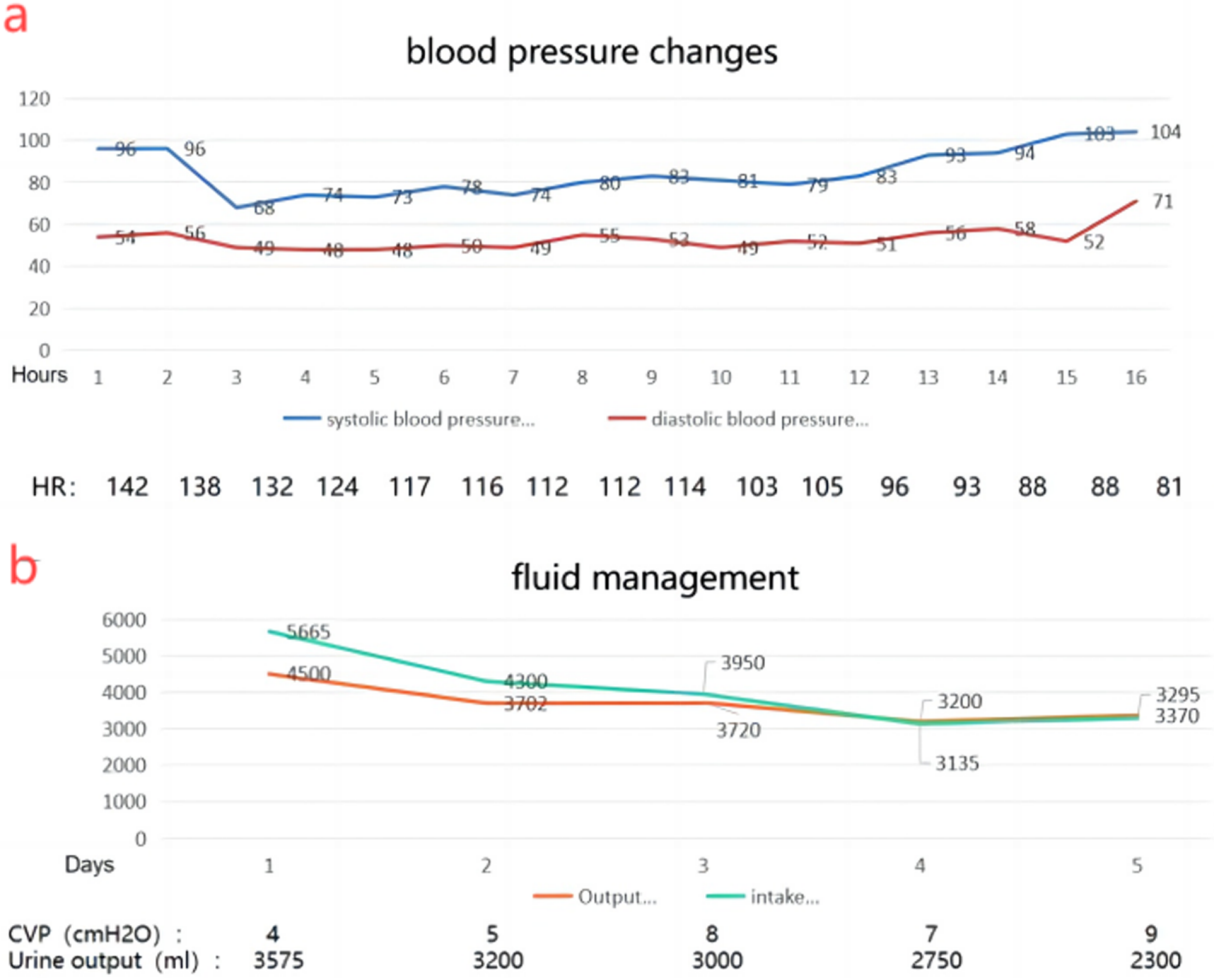
**(a)** changes in blood pressure 2 days after surgery suggest hypovolemic shock. **(b)** Changes in fluid intake and output after surgery.

Unfortunately, the patient experienced hypovolemic shock postoperatively, requiring fluid resuscitation, blood transfusion, and vasopressor therapy (Figure 6). On the 7th day, wound infection occurred, and based on bacterial culture results (Enterococcus avium) and susceptibility testing, meropenem, vancomycin, and levofloxacin were administered sequentially. The patient showed improvement and was discharged on the 25th postoperative day. Radiological surveillance was initiated with a baseline contrast-enhanced CT three months postoperatively, followed by contrast-enhanced CT every six months for the first two years and annually thereafter for 5–10 years. A 20-month follow-up showed no evidence of recurrence.

## 3 Discussion

PMP is a rare clinical syndrome, with over 95% of cases originating from the appendix and gastrointestinal tract. In the 1990s, Professor Sugarbaker (6) proposed the CRS combined with HIPEC combined modality for treating PMP, involving surgical resection of tumor tissue followed by intraperitoneal hyperthermic chemotherapy to eradicate residual microscopic foci and free tumor cells within the abdominal cavity, thus reducing tumor recurrence or prolonging the interval to recurrence. Results from multicenter retrospective studies have shown that patients undergoing CRS combined with HIPEC treatment may achieve a 10-year survival rate of over 60%, making this combined modality the preferred treatment approach for PMP (3, 4, 7).

However, nearly 30% of PMP patients experience recurrence following CRS combined with HIPEC (8), with studies indicating that a PCI > 20 is considered a risk factor for early recurrence, elevated preoperative CEA levels predict early recurrence and poorer survival rates in PMP, and higher CC scores correlate with a greater risk of recurrence (4, 9). Some scholars also suggest that regular postoperative systemic chemotherapy can reduce the risk of PMP recurrence (10). Current views suggest that systemic chemotherapy may be beneficial for patients with unresectable high-grade appendiceal adenocarcinoma, particularly in cases where tumor residue remains post-surgery. However, chemotherapy is generally not recommended for low-grade tumors, as they exhibit low cellular turnover and are typically resistant to such treatments (11). Recent advances in precision oncology and targeted therapies are being explored as we gain a better understanding of the genomic landscape of these tumors.

Although the spleen is a common metastatic site for malignant tumors, its metastasis typically occurs through hematogenous dissemination from primary or secondary tumors (12). However, both primary and recurrent metastasis of PMP to the spleen are rare, often presenting as diffuse intraperitoneal mucinous deposits, with lymphatic and hematogenous metastases being relatively infrequent (13). In our case, gelatinous material was observed upon splenic incision, and the exact metastatic route remains unknown, suggesting possible infiltration into the spleen or hematogenous dissemination.

In managing recurrent PMP with parastomal hernia, we propose several treatment strategies. Multidisciplinary team consultation is essential for thorough patient assessment, including cardiovascular and pulmonary evaluations to minimize perioperative risks. During abdominal wall repair, the choice of mesh is critical; we used Johnson & Johnson's UPA31015 (absorbable monofilament polyglecaprone-25 fibers and non-absorbable polypropylene monofilament fibers) mesh to reinforce the abdominal wall and prevent hernia recurrence. Postoperative care focused on reducing intraperitoneal fluid accumulation and managing intraoperative bleeding to avoid hypotension from decreased intra-abdominal pressure, necessitating abdominal binding and blood pressure monitoring. Tailored fluid and nutrition management was crucial for maintaining stable hemodynamics and promoting recovery. Effective pain management, including the use of rectus sheath block, was employed to enhance patient comfort. This case also illustrates the success of iterative CRS and HIPEC in treating recurrent PMP, even after initial suboptimal cytoreduction (CC2) and high-volume disease. Despite incomplete initial resection, the patient remained disease-free for four years, supporting the feasibility of repeat CRS and HIPEC in low-grade PMP with prolonged disease-free intervals. Residual disease, primarily in the upper abdomen and pelvis, was effectively managed during the second surgery, highlighting the potential for favorable outcomes with aggressive re-intervention, even following a CC2 resection.

## 4 Conclusion

This case presents a rare occurrence of recurrent low-grade appendiceal mucinous neoplasm with PMP, accompanied by parastomal hernia and splenic metastasis. When managing recurrent cases, preoperative assessment, intraoperative procedures, and postoperative monitoring are crucial. For patients with accompanying parastomal hernia, a multidisciplinary team approach and personalized treatment plans can enhance survival rates and improve prognosis. Prompt surgical removal of splenic metastasis is important to prevent further spread and is a key therapeutic approach. However, further research and practice are needed to optimize treatment strategies.

## Data Availability

The raw data supporting the conclusions of this article will be made available by the authors, without undue reservation.
